# Cost Analysis of Laparoscopic Low Anterior Resection vs. Transanal Endoscopic Microsurgery for Rectal Neoplasms

**DOI:** 10.3390/curroncol28030167

**Published:** 2021-05-11

**Authors:** Katerina Neumann, Nirmal Randhawa, Jason Park, David J. Hochman

**Affiliations:** 1 Division of General Surgery, Dalhousie University, 8-819 Victoria Building, VGH, 1276 South Park Street, Halifax, NS B2H 2Y9, Canada; 2 Faculty of Medicine, University of Manitoba, 750 Bannatyne Ave, Winnipeg, MB R3E 0W2, Canada; nirmal.randhawa@gmail.com; 3 Division of General Surgery, Vancouver General Hospital, 5th Floor—2775 Laurel Street, Vancouver, BC V5Z 1M9, Canada; jason.park1@vch.ca; 4 Division of General Surgery, University of Manitoba, St. Boniface Clinic, 343 Tache Avenue, Winnipeg, MB R2H 2A5, Canada; davehochman@hotmail.com

**Keywords:** TEM, TAMIS, transanal endoscopic microsurgery, LAR, low anterior resection, cost-analysis, rectal neoplasm

## Abstract

Despite the increasing application of transanal endoscopic microsurgery (TEM) for rectal lesions, the cost of the equipment may play a role in a hospital’s hesitancy to invest in the platform. This study compares the cost of TEM to laparoscopic low anterior resection (LAR). Patients who underwent laparoscopic LAR (*n* = 24) for rectal neoplasm between 2006 and 2014 were case-matched based on sex, age, comorbidities, lesion size and location to patients who underwent TEM at a busy secondary care urban hospital. Procedure-related costs and costs associated with readmissions for complications and related subsequent surgeries in the first 3 years were calculated. There were 42 hospital admissions for 24 LAR patients, totalling 326 hospital days. For 24 TEM patients, there were 25 hospital admissions, totalling 56 hospital days. Subsequent operations for LAR patients included 2 washout and diverting ileostomies (8%), 2 adhesionolysis (8%), 4 ventral hernia repairs (16%) and 11 ileostomy reversals (46%). In the TEM group, there was one operation for recurrence (4%). The mean cost of LAR, including all related hospital costs in the subsequent 3 years, was CAD 14,851 (95% CI: CAD 10,124–19,579). The mean cost of TEM was CAD 2449 (95% CI: CAD 2133–2767; *p* < 0.0001), with a savings of CAD 12,402 per patient. TEM for rectal neoplasm is associated with significantly lower hospital costs, which far outweigh the costs of acquiring and maintaining the technology.

## 1. Introduction

Transanal endoscopic microsurgery (TEM) has become the standard of care, when available, for the local excision of benign polyps and is considered an acceptable alternative to radical resection for select early rectal carcinomas. It has been shown to have superior results in achieving negative margins, less specimen fragmentation and lower local recurrence rates in comparison to traditional transanal local excision for both benign and malignant lesions [1,2,3]. TEM has also expanded the utility of local excision, permitting treatment of upper rectal lesions that are not accessible by traditional transanal techniques. Treatment of these upper rectal lesions that would otherwise require a formal low anterior resection (LAR) with TEM is much less morbid for the patient; in select cases, it has equivalent oncologic outcomes and is associated with much shorter hospital stays and fewer readmissions [4,5]. With these encouraging results, it is interesting to note that it has been slow to become available at many Canadian hospitals and many other countries. The cost of the equipment needed to perform TEM is not negligible and, thus, may play a role in a hospital’s hesitancy to invest in the TEM platform.

To date, there are no Canadian studies that look at the cost of TEM in comparison to laparoscopic or open low anterior resection. Maslekar and colleagues were able to show that the procedural costs of open radical resection for rectal neoplasms were more than 7 times that of TEM in the United Kingdom [6]. Cocilovo published cost data from the United States, comparing LAR and TEM on cases performed in the 1990s, showing that the average cost of TEM was USD 7775 in comparison to USD 34,018 for LAR [5]. The cost in these countries may be quite divergent from the Canadian experience. The Canadian health care system is a public system. Canadian hospitals are funded by their province based on productivity and case volumes. It is in the hospital’s best interest to keep running costs as low as possible while optimizing productivity. The Canadian hospital is burdened by the need to purchase the equipment necessary to perform the procedure. This includes the reusable, rigid platform, such as the Richard Wolf TEM proctoscope, along with the insufflations system, the 50-degree angled laparoscopic camera, and the specially designed angled laparoscopic instruments. This upfront financial burden, combined with maintenance costs, serves as a barrier to the technology becoming available at hospitals. In this study, we assess the hospital costs associated with laparoscopic LAR and any directly related admissions over a 3-year time period in comparison to the costs associated with TEM in a case-matched cost analysis of benign and early malignant rectal lesions.

## 2. Methods

All patients who underwent laparoscopic or laparoscopic hand-assisted LAR for either a benign polyp or good prognosis rectal cancer without threatened margins and were able to undergo a straight-to-surgery approach at a busy secondary care urban hospital in Manitoba between 2006 and 2014 were screened (*n* = 265). Patients who had undergone prior pelvic radiation or had recurrent or synchronous lesions or underlying IBD were excluded, leaving a population of 82 eligible LAR cases. They were case-matched based on sex, age, comorbidities, tumour size and tumour location to patients who underwent TEM during this time frame (*n* = 171). Medcalc case-matching software was utilized, where *p* > 0.05 was the cut-off to show no significant difference between matched pairs for all variables. We designated an allowable difference of 15 for age, 3 cm for size, and 5 cm for location, while exact matching was used for categorical variables, sex and ASA. A total of 24 cases were successfully matched. The initial hospital stay and additional costs associated with readmissions for complications, reversal of ileostomies, and surgery for late complications or recurrences in the first 3 years after the initial procedure were included in the cost analysis.

For cost calculations, we recorded details such as the use of specialized instruments intraoperatively (ligasure, staplers); the type, complexity and length of anesthetic; the length of the operation; the number of nursing and support staff during the case and for room turnover; the time spent by the patient in recovery; the time spent on the ward and/or in the intensive care unit; nursing and support staff in the ward/ICU; and all tests that were performed during the hospital stay as well as costs stemming from complications during the hospital stay and follow-up. Any complications that led to emergency department visits or readmissions were noted as well as any admissions for additional surgeries necessary as a direct result of the index surgery (e.g., ileostomy reversal, hernia surgery, surgery for recurrence). The cost calculations strictly reflect the operating cost to the hospital. They do not include the fees independently billed by the surgeon, anesthetist, or any other physician to the payer (the province of Manitoba) since these are not paid from the hospital budget. Please see Appendix A for complete details about the cost calculations.

Descriptive statistics are reported as counts and percentages for categorical variables, means for normally distributed continuous variables, and medians for non–normally distributed continuous variables. Baseline differences between treatment groups were examined using chi-square tests, Fisher’s exact test, Student’s *t*-test and the Wilcoxon rank-sum test, as appropriate. Differences were considered statistically significant when *p* < 0.05.

All surgeries were performed by a single colorectal fellowship-trained surgeon. Laparoscopic LAR cases were performed with laparoscopic clipping of the IMA, laparoscopic TME dissection and intracorporal anastomosis; laparoscopic hand-assist cases were performed with the use of a gel hand-port for mobilization of the left colon and splenic flexure, combined with open dissection of the TME plane and stapled colorectal anastomosis through a small lower midline or pfannenstiel incision. TEM cases were all performed using the Wolf TEM system using either a 12 or 20 cm anal tube, depending on the location of the lesion. Our practice includes a full thickness excision of the lesion and closure of the defect whenever possible.

## 3. Results

A total of 24 laparoscopic or laparoscopic hand-assisted LARs fitting the criteria were identified and case-matched against 24 TEM surgeries based on sex, age, comorbidities, tumour size and tumour location. The case-matching algorithm confirmed no significant differences between matched cases in any of the five variables. Patient and lesion characteristics are shown in Table 1. The number of invasive cancers in the LAR group (21/24, 87%) was significantly higher than in the TEM group (6/24, 25%), *p* < 0.0001.

There were 42 hospital admissions and 43 operations for the 24 LAR patients, totalling 326 hospital days that stemmed from the index operations over a 3-year period. In the 24 TEM patients, there were 25 hospital admissions and 25 operations, totalling 56 hospital days. Subsequent operations in the LAR group included 2 washout and diverting ileostomies (8%), 2 adhesionolysis (8%), 4 ventral hernia repairs (16%) and 11 reversal ileostomies (46%). In the TEM group, there was one additional operation for recurrence (4%).

Time spent in the OR and LOS was significantly different between the LAR and TEM groups (*p* < 0.0001). OR time was calculated from the time the patient entered the operating room to the time when the patient was transferred to recovery. The median OR time for LAR operations was 258 (IQR 237–280) min; for TEM operations, it was 118 (IQR 101–156) min. When calculating the cumulative OR time, including all subsequent operations, the median OR time per LAR patient was 300 (IQR 241–386) min; per TEM patient, it was 118 (IQR 101–157) min. The median length of hospital stay (LOS) for LAR operations was 7 (IQR 6–8) days; for TEM operations, it was 2 (IQR 2–2) days. When including the days spent in hospital during subsequent hospital admissions, the median number of hospital days was 10.5 (IQR 7–15) for LAR patients and 2 (IQR 2–2.5) for TEM patients. The postoperative complications were significantly lower in the TEM group than in the LAR group (*p* = 0.0002), as shown in Table 2. Some patients had more than one complication; however, only the highest on the Clavien Dindo scale is listed, per patient.

The mean costs (Canadian dollars) associated with the index operation and hospital stay for LAR patients and TEM patients are shown in Table 3. The cumulative cost for all hospital admissions stemming from the index operation is also depicted. The mean cost of each component of the total hospital stay, namely, the costs associated with the OR itself and the costs associated with ward care, diagnostics, and indirect hospital costs, for LAR is compared to TEM in Table 4. Each component is significantly more costly for LAR patients than for TEM patients.

## 4. Discussion

Our study shows that TEM is associated with significant cost savings in comparison to laparoscopic and laparoscopic hand-assisted low anterior resection for rectal neoplasms. For every case that is amenable to TEM, where LAR can be avoided, the hospital saves CAD 12,401.99. This does not include the additional cost savings to the provincial health care system for surgeon and anesthesia billing fees. It is a reflection of the direct savings to the hospital for operational costs and can, therefore, be directly compared to the hospital’s capital expenditures for gaining the equipment necessary to perform TEM.

The cost of the Wolf TEM equipment is approximately CAD 140,000 for the first set (quote from Vantage Endoscopy, 2017), and the cost of disposables and maintenance can be estimated at CAD 4000 yearly at a moderate volume center where the equipment is being used regularly. The life span of the equipment is a minimum of 15 years. The total costs associated with the equipment over a life span of 15 years would, therefore, be estimated at CAD 200,000. This cost would be made up after the first 17 cases, and the hospital would see a significant cost-benefit thereafter. The role of this study is not meant to comment on expected case volumes, credentialing and how hospital privileges should be determined but solely to demonstrate that even low case volumes still translate into hospital savings.

As already established, there are very few comparative studies published that show the cost difference between TEM and LAR. None of the available data takes into consideration the hospital’s point of view, although it is the hospital that often has to make the capital purchase of the equipment. The approach taken in this study is novel as it focuses on the operational costs of the hospital and shows a direct cost-benefit for the party that is required to make the upfront investment. It is also novel in that it includes the costs associated with subsequent hospital care for these patients, as this is significant in determining the true ramifications of the index surgery and is often overlooked in cost analyses.

It is important to consider the other surgical options for these lesions. Many surgeons perform open LAR for rectal neoplasms. Although this study strictly compares TEM to laparoscopic or laparoscopic hand-assisted LAR, we can make reasonable assumptions regarding the cost comparison to open surgery based on published data that compares laparoscopic colectomy to open colectomy. There are numerous studies that show that laparoscopic colectomy results in significantly lower overall hospital costs compared to open colectomy [7] and others that show it is at least as cost-beneficial as open surgery, including a randomized trial by Braga and colleagues [8,9]. From this, we can extrapolate that the cost-benefit shown in our study would also apply when comparing TEM cases to open LAR. TAMIS (transanal minimally invasive surgery) is another well-established technique similar in approach to TEM, using a disposable proctoscope and standard laparoscopic instruments to create a pneumorectum. This cost analysis is not generalizable to TAMIS, which may have lower up-front costs than TEM but higher longitudinal expenditures due to the use of disposables. Another emerging technique for removing rectal lesions, using a minimally invasive technique with low morbidity, is the use of endoscopic submucosal dissection; however, good quality data comparing the oncologic outcomes with this technique to TEM is still lacking. As this technique becomes more established, carrying out a cost analysis comparing TEM and ESD would be an interesting future consideration.

There are some notable limitations to this study. Most importantly, the study is based on the Canadian public health care system, which is not easily extrapolated to other countries. Furthermore, although we perform high-volume TEM and are based out of a quaternary care teaching hospital, the TEM equipment is housed at a secondary care affiliated community hospital; thus, all cases included were specifically from this community site. Lack of residents and the fact that the surgeon is not primarily based out of the community hospital may have led to longer LOS for LAR cases (median 7 days) than seen at other high-volume teaching hospitals that have implemented an ERAS (enhanced recovery after surgery) program. This may have led to an overestimation of cost for LAR. However, most of our TEM patients were discharged on the day following surgery, also adding to cost, where many other centers across Canada have been shown to safely offer TEM as day surgery. Thus, the cost for TEM may also be correspondingly overinflated. Nevertheless, this model serves as a business model for an individual corporation, namely, the health care facility, and our data can therefore be generalized to even the community setting. Because this study shows such dramatic cost savings, we feel that the information may be useful to other centers internationally when deliberating the purchase of the equipment. Furthermore, insurance companies and other health care funding agencies in some countries, including the United States of America, are still struggling to determine the appropriate remuneration for the procedure and may find this data useful or contributory in determining the value of a TEM resection.

Limitations pertaining to the collection of cost data also need to be noted. The costs of certain items were estimated in this study. The cost of dressings and other supplies used in PACU and the wards, such as reinsertion of a foley catheter, NG tube, and IV lines, etc., were estimated for a typical LAR case and a typical TEM case (per day, if applicable). Medications were estimated as a daily average per patient. We realize there can be considerable variance in the cost of home medications for each patient as well as variation in postoperative analgesia per shift for laparoscopic LAR cases in comparison to TEM cases, and this variation per shift was not captured by our study.

Another very important limitation lies in the fact that although this was a case-matched study, we were unable to case-match for histology and stage. There were many more malignant lesions in the LAR group than in the TEM group. This is an important consideration; however, in our opinion, this should not significantly affect the cost data. This study compared two different technical approaches, and our approaches were always consistent regardless of indication. Full-thickness excisions are routinely performed at our institution for all TEM cases regardless of whether they are benign or malignant; thus, the technique, the remainder of the hospital stay and postoperative early and late complications should not be affected by the underlying histology. The same holds true for all LAR cases, whether performed for benign or malignant lesions—a full oncologic resection, including nodal harvest following the TME plane, is always performed. The main drivers of cost in this analysis include the cost of the OR itself and the cost associated with the postoperative stay, subsequent ED visits, readmissions or operations. These variables are most importantly influenced by the type of operation performed and the complication profile of that procedure. Our complication profile was quite typical for both LAR and TEM cases. The only influence histology or stage could have on cost would be the costs associated with complications that may be more common in cancer patients or caused by recurrences. Such things would include complications influenced by neoadjuvant or adjuvant cancer treatments or patient frailty caused by tumour burden. We have controlled for this in our study. None of the cancer patients included in this study had neoadjuvant radiation or chemotherapy. There were no subsequent hospital stays or operations that were directly related to adjuvant cancer treatments. The patients were all case-matched for comorbidities, and only good prognosis cancers that were appropriate for a straight-to-surgery approach were included to eliminate frailty as a result of tumour burden. The majority of subsequent operations were for reversal of ileostomy or hernia repair. Although case-matching for histology and stage may have strengthened our results, this is simply not feasible even for a high-volume center since, with the availability of TEM, very few LAR operations are performed for benign or very early histopathology. We feel that in this data set, we have minimized the effect of histology and stage on cost, and the differences shown in our study are valuable from a cost business model perspective.

Finally, the numbers are small, but given the marked differences, even a small sample size demonstrates the significant cost savings to the hospital.

## 5. Conclusions

TEM is a widely accepted technique for local excision of rectal neoplasms, showing great advantages in terms of quality of specimen over standard local excision, and has the potential to access lesions in the upper rectum that would otherwise require a morbid formal radical resection. This modality is hindered from becoming widely available due to the burden of the capital investment required to purchase the equipment. This study demonstrates the significant cost-savings to the hospital over time, even with the purchase of the TEM equipment and the ongoing maintenance costs, when LAR can be avoided. It aims to show the importance and feasibility of breaking down cost barriers in order to provide the best possible and least invasive care for patients.

## Figures and Tables

**Table 1:** Patient and lesion characteristics.

Characteristic	LAR (*n* = 24)	TEM (*n* = 24)	*p*
mean age	60.7 (40–85)	59.8 (32–79)	0.812
gender male	12 (50%)	12 (50%)	1
ASA 1	3 (13%)	3 (13%)	1
ASA 2	19 (79%)	19 (79%)	1
ASA 3	2 (8%)	2 (8%)	1
mean size *	3.3 (1–7)	2.9 (0.04–5.5)	0.085
mean location **	10.8 (4–20)	9.7 (3–16)	0.323
Histopathology			<0.00001
Adenoma +/− HGD	3 (12%)	18 (75%)	
Adenocarcinoma	21 (88%)	6 (25%)	
Stage 1	6 (25%)	6 (25%)	
Stage 2	5 (21%)		
Stage 3	10 (42%)		

* largest diameter; ** distal extent of lesion measured from anal verge.

**Table 2:** Summary of complications.

Complications	Clavien Dindo Classification	LAR (*n* = 24)	TEM (*n* = 24)	*p*
Any Complication		12 (50%)	1 (4%)	0.0002
Minor Complication	Grade I—minor deviation	6 (25%)	0 (0%)	
	Grade II—requiring pharmacotherapy	2 (8%)	1 (4%)	
	Total minor complications	8 (33%)	1 (4%)	0.009
Major Complication	Grade III—requiring intervention			
	a. General anesthetic—no			
	b. General anesthetic—yes	2 (8%)	0 (0%)	
	Grade IV—requiring ICU			
	a. Single organ system	1 (4%)	0 (0%)	
	b. Multiorgan system			
	Total major complications	3 (12%)	0 (0%)	0.08
Mortality	Grade V—death	1 (4%)	0 (0%)	0.32

**Table 3:** Mean cost comparison LAR vs. TEM.

Variation	Index Hospital Admission					Cumulative: All Hospital Admissions Per Patient		
	OR Cost	Ward/ICU Cost	Diagnostics	Indirect Cost	Total Cost	Admissions	Surgeries	Cumulative Cost
LAR	3147.16	2589.42	341.08	3355.25	9432.90	1.75	1.79	14,851.36
TEM	890.83	324.38	18.56	1127.50	2361.26	1.04	1.04	2449.36

Cost expressed in CAD.

**Table 4:** Summary of cost savings.

Variation	LAR	TEM	*p*	Cost Savings
Index hospital admission				
OR cost	3147.16	890.83	<0.0001	2256.33
Ward/ICU cost	2589.42	324.38	<0.0001	2265.04
Diagnostics	341.08	18.56	<0.0001	322.52
Indirect cost	3355.25	1127.50	<0.0001	2227.75
Total cost	9432.90	2361.26	<0.0001	7071.64
Cumulative hospital admissions				
OR costs	3942.21	925.80	<0.0001	3016.42
Ward/ICU costs	4330.88	334.25	<0.0001	3996.63
Diagnostics	727.52	19.03	<0.0001	708.49
Indirect costs	5850.75	1170.29	<0.0001	4680.46
Total cumulative costs	14,851.36	2449.36	<0.0001	12,401.99

Cost expressed in CAD.

## Data Availability

The data presented in this study are available on request from the corresponding author. The data are not publicly available due to patient privacy and confidentiality.

## Appendix A. Cost Calculations

The cost of each surgery was calculated as the cost of all the disposable equipment used during the case (reprocessing of reusable equipment was not included in our calculation), including the use of specialized instruments such as the ligasure, bowel staplers etc. The cost of the anesthetic was calculated based on type of anesthetic and time it was administered (in minutes), as well as the cost of disposables used by anesthesia for each type of case, including regional anesthesia when given, placement of art-line etc. The cost of the operating room (OR) took into consideration the time needed for the case including turn-over and the number of support staff required including nursing staff and support staff for cleaning the room. The cost of time spent in recovery took into consideration the cost of nursing staff for time spent in recovery as well as average disposables per type of case.

The cost of time spent on the ward or ICU was calculated in number of 8-h nursing shifts. The cost of nursing staff per shift was calculated depending on the type of surgery performed (taking into consideration higher needs for post-LAR patients than for post-TEM patients per shift). The disposables per shift (dressings, iv lines, catheters, etc.), average medications per shift, the support staff needed per shift (physiotherapy, nutrition, ward clerk, health care aids, etc.) were all calculated as an average cost per shift, based on the type of surgery performed. The ward cost calculations also included an individualized list of all diagnostics per patient and the cost of each, including laboratory, diagnostic imaging, cardiac investigations.

Furthermore, we calculated the daily fixed cost (indirect cost) per patient for non-variable hospital costs such as maintenance, admissions, dietary and administrative costs.
